# An unusual case of *Scedosporium apiospermum* fungaemia in an immunocompetent patient with a left ventricular assist device and an implantable cardiac device

**DOI:** 10.1099/acmi.0.000148

**Published:** 2020-06-25

**Authors:** Shireen Yan Ling Tan, Shimin Jasmine Chung, Teing Ee Tan, Louis Teo, Ban Hock Tan, Yen Ee Tan

**Affiliations:** ^1^​ Department of Microbiology, Singapore General Hospital, Academia, 20 College Road, Singapore 169856, Singapore; ^2^​ Department of Infectious Diseases, Singapore General Hospital, Academia, 20 College Road, Singapore 169856, Singapore; ^3^​ National Heart Centre Singapore, 5 Hospital Drive, Singapore 169609, Singapore

**Keywords:** ventricular assist device, cardiac device, blood, fungaemia, immunocompetent, *Scedosporium apiospermum*

## Abstract

Left ventricular assist device (LVAD)-related infections are a leading cause of morbidity and mortality, with fungal infections being particularly difficult to manage. We report a case of an immunocompetent 39-year-old male with an LVAD and an implantable cardiac device (ICD) who developed fatal *Scedosporium apiospermum* fungaemia. To the best of our knowledge, this is the first reported case of LVAD-related *S. apiospermum* fungaemia.

## Introduction

Left ventricular assist devices (LVADs) are implantable mechanical circulatory pumps that are indicated for patients with end-stage heart failure as bridge-to-transplant or as destination therapy [1, 2]. LVAD systems consist of a pump connected to the heart via inflow and outflow cannulas, and a driveline tunnelled percutaneously from the pump out of the body through an exit site to an external power source [2].

Infectious complications following LVAD implantation are a leading cause of morbidity and mortality [3]. Although most infections involve the percutaneous driveline, other components of the LVAD system, including the pump and pump pocket, can also be affected [1, 4, 5]. Bacterial infections are by far the most common, followed by fungal infections, which account for 6.6–23 % of LVAD-related infections [5]. *Candida* species are the most commonly isolated fungal pathogen, but other fungal pathogens, such as *Aspergillus* species, have also been isolated [5, 6].

Here, we report an unusual case of *Scedosporium apiospermum* fungaemia in an immunocompetent patient with an LVAD and an implantable cardiac device (ICD) *in situ*. To the best of our knowledge, this is also the first case of *S. apiospermum* LVAD-related infection.

## Case report

A 39-year-old male was diagnosed with left ventricular non-compaction cardiomyopathy in 2014, and an ICD was put in place in 2016. In June 2017, he underwent an uncomplicated HeartWare (Heartware International, Inc.) LVAD implantation.

In June 2018, he dropped his LVAD controller while playing badminton, tugging on his LVAD driveline. He subsequently developed methicillin-susceptible *Staphylococcus aureus* driveline infection. This was treated with an 11-day course of intravenous cloxacillin followed by oral cloxacillin suppression.

While on cloxacillin suppression, he presented in July 2018 for evaluation of a 1-week history of exertional dyspnoea and firing of his ICD due to ventricular tachycardia.

**Fig. 1.**
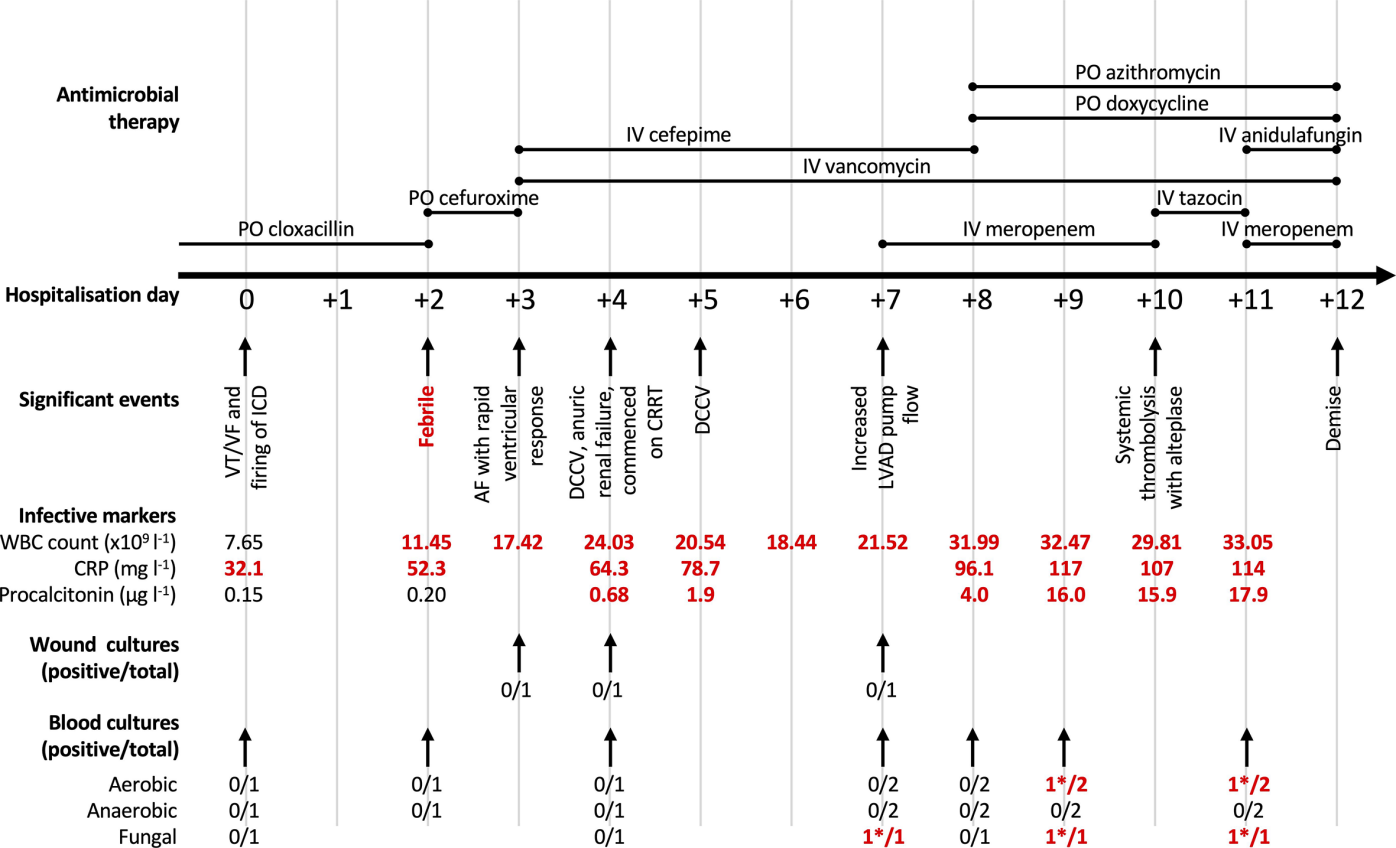
Clinical progress. This figure highlights significant events, antimicrobial therapy administered, infective markers, and wound and blood cultures sent during this admission. *All positive blood cultures grew *S. apiospermum*. PO, oral; IV, intravenous; VT, ventricular tachycardia; VF, ventricular fibrillation; ICD, implantable cardiac device; AF, atrial fibrillation; DCCV, direct current cardioversion; CRRT, continuous renal replacement therapy; LVAD, left ventricular assist device; WBC, white blood cell; CRP, C-reactive protein.

At presentation, he was afebrile, with no localizing signs of infection; his driveline was well healed, and apart from a slight increase in C-reactive protein (CRP) at 32.1 mg l^−1^, his other infective markers were within normal limits, as shown in Fig. 1. On hospital day 2, he developed a fever with a temperature of 38.2 °C and a new pustular lesion near his driveline exit site was noted. He was empirically started on cefepime and vancomycin. On hospital day 3, he developed persistent atrial fibrillation with rapid ventricular response, requiring direct current cardioversion (DCCV) on hospital days 4 and 5. This was associated with progressive leukocytosis and rising CRP and procalcitonin levels, despite cefepime and vancomycin therapy (Fig. 1). A contrasted computed tomography of the chest, abdomen and pelvis performed on hospital day 4 did not reveal any infective foci, and no vegetations were seen on the transthoracic echocardiograms performed on hospital days 5 and 8.

Despite broad-spectrum antibiotics, there was progressive clinical deterioration, as detailed chronologically in Fig. 1. On hospital day 4, he developed anuric renal failure requiring continuous renal replacement therapy (CRRT). On hospital day 7, his LVAD pump flow had increased, which was suggestive of ongoing sepsis that was not well controlled. This correlated with the biochemical markers, which showed uptrending inflammatory markers. Unfortunately, serial blood cultures collected through the early course of the admission remained negative, precluding a microbiological diagnosis. Over the next few days, he developed pump thrombosis, which manifested as cardiogenic shock with multi-organ failure, haemolysis (LDH >15 000 U l^−1^, haptoglobin <0.10 g l^−1^) and abnormal LVAD parameters with elevated pump power of 7.3 watts and peak pump flow of 10 l min^−1^. LVAD pump exchange was deemed inappropriate due to the high operative mortality risk and ongoing sepsis with risk of reinfection of a new LVAD pump. On hospital day 10, systemic thrombolysis was attempted using alteplase without success, as there was no resolution of cardiogenic shock and haemolysis, with persistently elevated LVAD pump power and flow. He continued to deteriorate and later expired on hospital day 12.

During the admission, serial LVAD driveline wound swabs for bacterial cultures returned negative. Multiple blood cultures were collected in BD BACTEC Plus Aerobic/F (‘Aerobic’), BD BACTEC Plus Anaerobic/F (‘Anaerobic’) and BD BACTEC Myco/F Lytic (‘Fungal’) bottles (BD Diagnostic Systems) (Fig. 1). These blood culture bottles were incubated in BD BACTEC FX blood culturing instruments (BD Diagnostic Systems) as per our laboratory’s standard protocol. In total, five blood culture bottles flagged positive, indicating growth detected by the blood culturing instrument, from specimens collected on hospital days 7, 9 and 11 (Fig. 1). Of these, one blood culture flagged positive just prior to his demise, and four blood culture bottles only flagged positive after his demise.

**Fig. 2.**
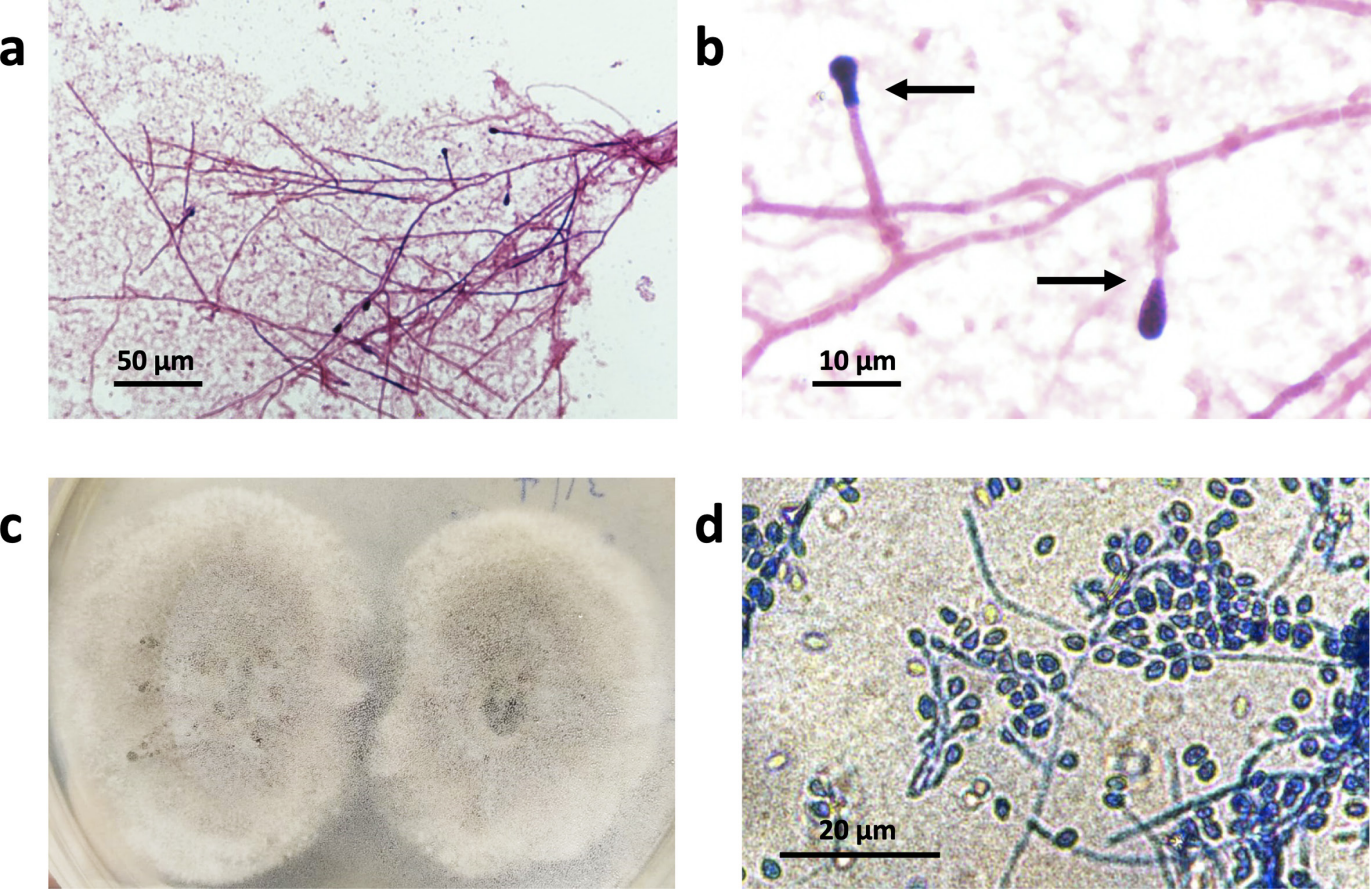
*S. apiospermum*. (a) Upon detection of growth in a blood culture bottle, Gram staining of broth from the positive blood culture bottles was performed. A representative Gram stain of broth from a positive blood culture at the point of growth detection, after 4 days of incubation in the BACTEC FX blood culturing instrument, is shown here. (b) Same as (a), but at higher magnification. Septate hyphae and conidia with truncated bases are observed (arrows). (c) Broth from the positive blood culture bottles was inoculated onto potato dextrose agar and incubated at 30 °C, obtaining fungal isolates with a house mouse grey morphology. The fungal isolates here were obtained after 5 days of growth. (d) Lactophenol cotton blue staining of the fungal isolate in (c) was performed, showing the characteristic septate hyphae and conidia arrangement of *S. apiospermum*. The conidia seen here appear to be more prolific than in (a) due to the different media used.

Gram stains of broth from the positive blood culture bottles showed septate hyphae and conidia with truncated bases. Broth from the positive blood culture bottles was inoculated onto potato dextrose agar and incubated at 30 °C, obtaining fungal isolates with a ‘house mouse’ grey morphology. Macroscopic and microscopic findings are presented in Fig. 2. Internal transcribed spacer (ITS) sequencing was carried out on the isolated colonies as described previously [7–9]. Briefly, fungal DNA was extracted and PCR was carried out using primers ITS1 (5′-TCCTCCGCTTATTGATATGC-3′) and ITS4 (5′-TCCGTAGGTGAACCTGCGG-3′) [7]. The amplified product was then sequenced using the same primers. The obtained DNA sequences were then blasted against the National Center for Biotechnology Information (NCBI) GenBank sequence database. The identity was determined as *S. apiospermum* complex. This is consistent with the phenotypic features.

Antifungal susceptibility testing was performed using a Sensititre YeastONE YO10 plate (Thermo Fisher Scientific). The *S. apiospermum* complex isolated had the following minimum inhibitory concentrations (MICs): anidulafungin 8 µg ml^−1^, micafungin≥8 µg ml^−1^, caspofungin ≥8 µg ml^−1^, posaconazole 0.5 µg ml^−1^, voriconazole 0.25 µg ml^−1^, fluconazole 16 µg ml^−1^ and amphotericin B 8 µg ml^−1^.

## Discussion

*S. apiospermum*, a ubiquitous environmental mould, can cause a wide range of clinical syndromes in both immunocompetent and immunocompromised hosts [10–12]. Here, we have described a fatal case of *S. apiospermum* fungaemia in an immunocompetent patient with an LVAD and an ICD *in situ*. In this patient, we postulate that the most likely route of acquisition was cutaneous inoculation of *S. apiospermum* onto the driveline exit site following local trauma, with subsequent haematogenous dissemination. To the best of our knowledge, this is the first described case of LVAD-related *S. apiospermum* fungaemia, and also the first described case of *S. apiospermum* fungaemia in Singapore.

Infectious complications following LVAD placement are common. Localized infections associated with the percutaneous driveline are the most common, but disseminated infections can also occur [1]. Patients with LVAD and disseminated infections may sometimes present atypically with minimal infective symptoms and delayed or diminished febrile responses [13], and alarms triggered by the implantable cardiac devices may be the only sign of infection. This is illustrated by this patient, whose presenting complaint was for evaluation of arrhythmia in association with firing of his ICD device, and who only developed fever on hospital day 2. Despite broad-spectrum antibiotics, there was progressive LVAD dysfunction (high pump power and pump flow) and thrombosis, suggesting uncontrolled sepsis, which in this case was contributed by *S. apiospermum* fungaemia. This case highlights that when alarms are triggered by the ICD with no objective cause for cardiac dysfunction, infectious complications should be considered [14].

In LVAD-related infections, bacterial pathogens are more commonly implicated than fungal pathogens, and of the fungal pathogens, *Candida* spp. are most often isolated [5]. As such, LVAD-related infections with unusual pathogens such as *S. apiospermum* are particularly difficult to diagnose and treat. Firstly, the clinical suspicion for such unusual pathogens is low. If a fungal LVAD-related infection is suspected, empirical therapy is often targeted at *Candida* spp., using either fluconazole or an echinocandin – neither of which are active against *S. apiospermum*. Secondly, obtaining a microbiological diagnosis is more challenging, given the low diagnostic yield for fungal pathogens [15]. Even if a positive fungal culture is obtained, a period of days to weeks may be required to identify the isolate and carry out antifungal susceptibility testing, which will contribute to the delay in administering appropriate antifungal therapy. Furthermore, positive fungal cultures are often associated with heavy fungal burden and advanced disease, and the diagnosis of such fungal LVAD-related infections is often made ante- or post-mortem. Lastly, eradication of fungal infection is challenging without device removal [16].

Whilst this is the first reported case of LVAD-related *S. apiospermum* infection, *S. apiospermum* infections have previously been reported in patients with other cardiac or intravascular devices [17, 18]. The prognosis in these previous reports is poor; a previous review of 12 cases of *S. apiospermum* and *Lomentospora* (formerly *Scedosporium*) *prolificans* infection related to cardiac or intravascular devices found only two patients who survived, both of whom had been treated with voriconazole and surgery [17]. Extrapolation from these previous reports would suggest that *S. apiospermum* LVAD infection carries a grim prognosis, especially if surgery for device removal is not feasible due to the technical challenges and associated peri-operative morbidity and mortality. The potentially life-threatening nature of LVAD-related infections with unusual pathogens (such as *S. apiospermum*) underscores the need for patient education on safe living and appropriate LVAD care to minimize unexpected environmental exposures and undesirable infective complications.

From an epidemiological point of view, *S. apiospermum* infection in this patient is unusual and unexpected. *S. apiospermum* is commonly found in temperate climates, but less frequently in tropical climates [10, 11, 19], such as Singapore’s. This is the only case of *S. apiospermum* fungaemia that has been seen in our institution in the past decade, with the majority of other cases identified during this time period being localized infections. In the local setting, the isolation of moulds in blood culture would more commonly be due to *Fusarium* spp. or *Talaromyces marneffei* fungaemia, and *Scedosporium* spp. may not be considered in the differentials due to the low incidence. However, the astute microbiologist might be able to recognize the characteristic appearance of *Scedosporium* spp. hyphae and conidia with truncated bases on a Gram stain (Fig. 2a, b) and alert the treating physician to the possibility of *Scedosporium* spp. infection, potentially shortening the time to appropriate antifungal therapy.

The identification of *S. apiospermum* is vital in guiding treatment. *S. apiospermum* is closely related to *L. prolificans*, and microscopically, they may appear similar. Both *S. apiospermum* and *L. prolificans* have high MICs to many antifungal agents. However, *S. apiospermum* typically appears to be more susceptible to systemic antifungal agents than *L. prolificans* [11]. Of the available antifungal agents, voriconazole exhibits the greatest activity against both fungal pathogens [11]. Voriconazole monotherapy is recommended as first-line treatment for *S. apiospermum* infections, while voriconazole combination therapy with terbinafine is recommended for *L. prolificans* infection [11, 12, 20]. The mainstay of fungal identification in most routine clinical diagnostic laboratories is phenotypic identification of a positive fungal culture [12, 20, 21], which is a process that may take days to weeks. In future, emerging technologies such as direct detection from positive blood cultures or whole blood may allow a quicker diagnosis, benefiting patients with rapid clinical deterioration [22, 23].

In conclusion, this case highlights the difficulty of diagnosing and treating uncommon LVAD-related mould infections such as *S. apiospermum*. Given the high morbidity and mortality associated with LVAD-related infections, it is important to educate patients on safe living and LVAD care to minimize the risk of developing these infections. Additionally, there should be a low threshold to investigate for infections in immunocompetent patients with LVAD, especially if alarms are triggered by ICDs or LVADs with no apparent cause from cardiac dysfunction. There is a need to keep in mind the possibility of fungal pathogens as the causative agents, especially if the patient does not respond to antibacterial treatment, as different therapeutic approaches may be required to counter these pathogens.
